# Pacific contribution to decadal surface temperature trends in the Arctic during the twentieth century

**DOI:** 10.1007/s00382-021-05868-9

**Published:** 2021-07-22

**Authors:** Lea Svendsen, Noel Keenlyside, Morven Muilwijk, Ingo Bethke, Nour-Eddine Omrani, Yongqi Gao

**Affiliations:** 1 Geophysical Institute, University of Bergen and Bjerknes Centre for Climate Research, Bergen, Norway; 2 Nansen Environmental and Remote Sensing Center and Bjerknes Centre for Climate Research, Bergen, Norway; 3 Nansen-Zhu International Research Center, Institute of Atmospheric Physics, Chinese Academy of Science, Beijing, China

**Keywords:** Arctic, Pacific, PDO, Multidecadal variability, Teleconnections, NorESM

## Abstract

Instrumental records suggest multidecadal variability in Arctic surface temperature throughout the twentieth century. This variability is caused by a combination of external forcing and internal variability, but their relative importance remains unclear. Since the early twentieth century Arctic warming has been linked to decadal variability in the Pacific, we hypothesize that the Pacific could impact decadal temperature trends in the Arctic throughout the twentieth century. To investigate this, we compare two ensembles of historical all-forcing twentieth century simulations with the Norwegian Earth System Model (NorESM): (1) a fully coupled ensemble and (2) an ensemble where momentum flux anomalies from reanalysis are prescribed over the Indo-Pacific Ocean to constrain Pacific sea surface temperature variability. We find that the combined effect of tropical and extratropical Pacific decadal variability can explain up to ~ 50% of the observed decadal surface temperature trends in the Arctic. The Pacific-Arctic connection involves both lower tropospheric horizontal advection and subsidence-induced adiabatic heating, mediated by Aleutian Low variations. This link is detected across the twentieth century, but the response in Arctic surface temperature is moderated by external forcing and surface feedbacks. Our results also indicate that increased ocean heat transport from the Atlantic to the Arctic could have compensated for the impact of a cooling Pacific at the turn of the twenty-first century. These results have implications for understanding the present Arctic warming and future climate variations.

## Introduction

Arctic surface temperatures have been increasing for decades, and the Arctic is warming at a higher rate than the rest of the globe. In addition to the long-term centennial warming, Arctic surface temperatures and sea ice extent display multidecadal variability throughout the instrumental record (Day et al. 2012; Kay et al. 2011; Moritz et al. 2002; Overland et al. 2004; Polyakov et al. 2002, 2003). This variability has been attributed to the combination of external forcing and internal variability (Day et al. 2012; Delworth and Knutson 2000; Kay et al. 2011; Wang et al. 2007; Zhang 2015) but their relative importance remains unclear.

Earlier studies have identified contributions to multidecadal variability in Arctic surface temperature from anthropogenic climate forcing such as atmospheric greenhouse gas concentrations and aerosols, and natural climate forcing such as volcanic eruptions and variations in solar insolation (Fyfe et al. 2013; Kay et al. 2011; Soon 2005). When it comes to internal variability, a discussion is ongoing about the relative importance of the Atlantic (Chylek et al. 2009; Johannessen et al. 2016) and the Pacific (Screen and Deser 2019; Screen and Francis 2016; Svendsen et al. 2018; Tokinaga et al. 2017). The role of the Pacific for Arctic variability on decadal and longer timescales has received less attention than the Atlantic until recently.

The Atlantic influence on the Arctic on decadal to multidecadal timescales has been identified to be mainly through poleward ocean heat transport (OHT) through the Barents Sea and the Fram Strait (Årthun et al. 2012; Day et al. 2012; Smedsrud et al. 2013). Particularly for the Barents Sea during winter and spring, increased OHT reduces sea ice formation (Årthun et al. 2012) and warms the Arctic atmosphere from below. OHT variability from the Atlantic into the Arctic on these timescales is driven by wind and sea level pressure (SLP) variations (Bengtsson et al. 2004; Dickson et al. 2000; Goosse and Holland 2005; Muilwijk et al. 2018), but some studies also suggest it could be related to variability in the overturning circulation (Chylek et al. 2009; Day et al. 2012). Decadal to multidecadal variability in the North Atlantic Oscillation (NAO) is associated with variations in Arctic sea ice cover (Deser et al. 2000). The NAO can also drive variations in the strength of the Atlantic meridional overturning circulation impacting the poleward OHT into the Arctic leading to Arctic sea ice changes (Delworth and Zeng 2016; Delworth et al. 2016). The Atlantic can also impact Arctic climate through atmospheric teleconnections (Castruccio et al. 2019).

In recent years, decadal variability in the Pacific has been earning attention for not only its impact on global surface temperatures trends (Kosaka and Xie 2013, 2016; Trenberth and Fasullo 2013), but also the impact on Arctic surface temperature and sea ice extent (Ding et al. 2014b, 2018; Meehl et al. 2018; Screen and Deser 2019; Screen and Francis 2016; Svendsen et al. 2018; Tokinaga et al. 2017). Hartmann and Wendler (2005) identified a link between the Pacific and Arctic temperatures related to the 1979 shift of the Pacific Decadal Oscillation (PDO). This shift coincided with a deepening Aleutian Low in winter and spring leading to increased transport of warm moist air northwards in the eastern North Pacific. Anomalous atmospheric heat and moisture transport from the Pacific into the Arctic leads to convergence of atmospheric energy, increasing downwards longwave radiation and turbulent fluxes. The surface albedo feedbacks then cause increased absorption of downwelling shortwave radiation the following spring and summer (Graversen et al. 2011).

A stronger/weaker Aleutian Low can also create positive/negative interference between the background and anomalous midlatitude stationary wave pattern (wave number 1) during winter. This interference strengthens/weakens the upwards propagating planetary wave activity flux, which weakens/strengthens the stratospheric westerlies, weakening/strengthening the stratospheric polar vortex (Fletcher and Kushner 2011; Hu et al. 2018). During winter, a weakening stratospheric polar vortex in the Arctic can lead to downward stratosphere-troposphere coupling (Ambaum and Hoskins 2002; Haynes 2005), with warm stratospheric temperature anomalies reaching the Arctic surface through subsidence induced adiabatic heating (Hurwitz et al. 2012; Svendsen et al. 2018).

In addition to the impact on the Arctic from advection and the interaction with the polar vortex, the variability of the Aleutian Low works as a boundary condition constraining Arctic atmospheric circulation and the variability of the Arctic Oscillation (Sein et al. 2014). The Pacific Ocean also impacts Arctic temperature and sea ice through transport of Pacific Water through the Bering Strait (Woodgate et al. 2010), but the total OHT here is more than an order smaller than from the Atlantic Ocean (Muilwijk et al. 2018; Woodgate et al. 2006).

Decadal variability in the Pacific has been linked directly to the early twentieth century Arctic warming that took place from around 1910 to the 1940s (Svendsen et al. 2018; Tokinaga et al. 2017). Specifically, Svendsen et al. (2018) found that decadal variability in the Pacific related to the phase change of the PDO from negative to positive and a deepening Aleutian Low could explain around 50% of the early twentieth century Arctic surface warming. The question remains as to whether decadal variability in the Pacific contributed to decadal trends of Arctic surface temperature during the rest of the twentieth century, after the warm peak in the 1940s. The present study is a follow-up study of Svendsen et al. (2018).

In this study, we investigate how variability in Pacific sea surface temperatures (SSTs) can impact surface temperature and atmospheric circulation in the Arctic on multidecadal timescales, and we identify how much of the multidecadal variability in Arctic surface temperature during the twentieth century can be explained by Pacific variability. Understanding how and to what degree Pacific variability impacts Arctic surface temperature trends under the present Arctic warming has implications for understanding future changes in the Arctic and for decadal predictions of Arctic climate.

In the following, we will first describe the data and methods we used for our analysis (Sect. 2). Then we will examine the statistical relation between the Pacific and Arctic surface temperature covering the twentieth century and investigate the Pacific impact on the Arctic for four different periods spanning the past century (Sect. 3). Following a discussion on possible interactions with multidecadal variability in the Atlantic and external forcing, we consider some regional differences in the Arctic response to Pacific variability and discuss uncertainties with the model and experimental design in this context (Sect. 4). A summary of our results (Sect. 5) concludes the paper.

## Data and methods

**Fig. 1:**
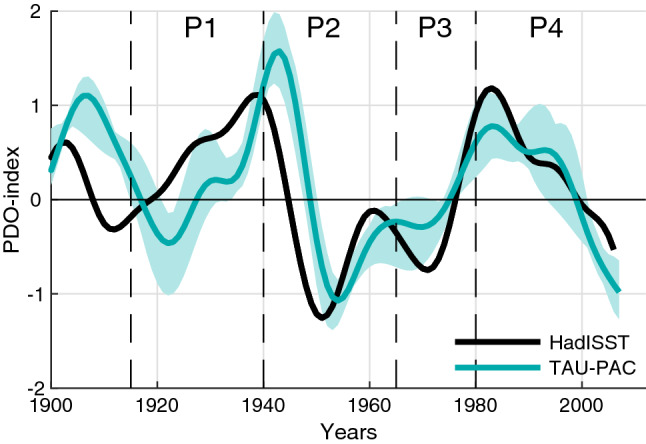
ONDJF PDO-index for HadISST (black solid line), and TAUPAC (green line). Green shading shows the ensemble spread for TAUPAC. The dashed vertical black lines indicate the separation of the observed warming and cooling periods P1-P4

To investigate the Pacific influence on Arctic surface temperature trends during the twentieth century we have performed two experiments with the Norwegian Earth System Model (NorESM). NorESM is a fully coupled earth system model. Here we use the Coupled Model Intercomparison Project 5 (CMIP5) version NorESM1-ME (see Bentsen et al. (2013) for more details). Our control experiment (CNTRL) consists of a six member ensemble of fully coupled historical simulations including all transient external forcing, as the historical CMIP5 simulations (Taylor et al. 2012). The ensemble members differ only in their initial conditions. The initial conditions are taken from a pre-industrial control simulation at 10-year intervals. Our second experiment, TAUPAC, also consisting of six ensemble members, is identical to CNTRL except that it is partially coupled over the Indo-Pacific Ocean by overwriting daily momentum flux anomalies in the ocean model with reanalysis interpolated to the model grid. The momentum flux ($\tau$) that the ocean model receives from the atmosphere model can be written as follows: $\tau ={\tau }_{C}+{\tau }_{A}$, where the subscripts C and A represent daily climatology and daily anomalies, respectively. In TAUPAC, the modification of the ocean model over the Indo-Pacific is as follows: ${\tau =\tau }_{C}^{m}+{\tau }_{A}^{r}$, where the superscripts m and r indicate the momentum flux from the model and from reanalysis, respectively. The Indo-Pacific domain covers the Indo-Pacific Ocean from 25° S to 60° N at the Bering Strait. We have a tapering region of linear weighting of 5° latitude outside these boundaries, so north of 65° N and south of 30° S the model is fully coupled. The longitudinal boundaries of the Indo-Pacific domain are given by the coastlines. By using this method of partial coupling, we synchronize the Indo-Pacific Ocean dynamical variability to the observed one, while simultaneously maintaining the thermodynamic atmosphere–ocean coupling in the model (Ding et al. 2014a). Specifically, we reproduce the observed phasing of the PDO (Fig. 1) and ENSO events (Svendsen et al. 2018).

In our TAUPAC experiment, we use the momentum flux product from the twentieth century reanalysis (20CR) from NOAA-NCAR which is the longest reanalysis at the moment (Compo et al. 2011). Both ensembles cover the period 1871–2012, the length of the NOAA-NCAR twentieth century reanalysis. Since both CNTRL and TAUPAC are based on the CMIP5 version of the model, historical external forcing only exists until 2006. For the last years of the simulations, we use the Representative Concentration Pathways 4.5 (RCP4.5) scenario for external forcing. Over this 7-year period there is little difference among the standard RCPs (Schwalm et al. 2020), and the related uncertainties in climate projections are small (Hawkins and Sutton 2009). Thus, we do not expect that our results would differ significantly if we had instead used the RCP8.5 scenario. Because of data quality in the beginning of the reanalysis and possible initialization issues to the momentum flux implementation related to the spin-up of the Pacific Ocean that could lead to a lag in Pacific Ocean variability (see Methods section in Svendsen et al. 2018), we disregard the first decades of the simulations and only analyze the period 1900–2012.

By comparing TAUPAC and CNTRL we can isolate the externally forced signal from that forced by Pacific variability, keeping in mind that the ensemble means will also reflect internal variability due to the ensemble size. A possible external forced signal of the Pacific momentum flux variability is not taken into account; we assume any such signal to be relatively weak as PDO and ENSO variability are believed to be internally forced (Newman et al. 2016). In NorESM specifically, there is no notable impact of external forcing on the PDO (See Supplementary Fig. 2a in Svendsen et al. 2018). For a more detailed description and evaluation of TAUPAC see the Methods section of Svendsen et al. (2018).

In addition to the historical ensembles, we analyze an 800-yr preindustrial control simulation (piCNTRL) of the same version of NorESM to further assess statistical robustness of our results and the consistency of our experimental design. The piCNTRL has constant greenhouse gas concentrations and aerosol emissions set to 1850 values according to CMIP5 protocol (Taylor et al. 2012).

For our analysis we are interested in Arctic and Pacific temperatures and atmospheric circulation patterns. We define the Arctic as north of 70° N, to exclude the area where the momentum flux anomalies are prescribed. The Arctic surface temperature index is the area-averaged surface temperature in this region. We define the PDO-index as the first EOF of monthly SST over the North Pacific from 20° to 65° N (Mantua et al. 1997). The Aleutian Low variability is quantified by the North Pacific Index (NP-index), which is defined as sea level pressure (SLP) averaged over the region 30–65° N and 160° E–140° W (Trenberth and Hurrell 1994). We also use a tropical Pacific index (TP-index) defined as the area-averaged SST in the region 25° S–25° N and 180° E–90° W.

As in Svendsen et al. (2018), we focus here on the role of the Pacific since TAUPAC can simulate observed variations in this basin. However, the possible influence of Atlantic SST and OHT through the Barents Sea Opening is also evaluated in our simulations. We define the Atlantic Multidecadal Variability (AMV) index by the area-weighted average North Atlantic SST from the equator to 70°N and 60–0°W. OHT generally varies due to changes in both ocean temperature and volume transport, and its absolute value depends on the chosen reference temperature (Schauer et al. 2004). The OHT through the Barents Sea Opening is here calculated as a net transport across the full strait using a reference temperature of *T*_*ref*_  = 0 °C, as commonly used in the oceanographic community and is close to the temperature of cold waters exiting the Barents Sea into the deep Arctic Ocean.

For comparison with observations we use HadISST (Rayner 2003) for SST, and GISTEMP (Hansen et al. 2010) and Nansen-SAT (Kuzmina et al. 2008) for surface temperature. The Nansen-SAT data covers the period 1900–2006, so for the years 2007–2012, GISTEMP is used. For SLP, we have used NOAA-NCAR twentieth century reanalysis (Compo et al. 2011) and the monthly NP-index retrieved from https://climatedataguide.ucar.edu/climate-data/north-pacific-np-index-trenberth-and-hurrell-monthly-and-winter (Trenberth and Hurrell 1994). We have also utilized the NSIDC gridded sea ice fraction data (Walsh et al. 2015, 2017). As an estimate of the historical OHT from the Atlantic Ocean through the Barents Sea Opening we use output from an ocean-sea ice-only twentieth century simulation of NorESM forced by an adjusted NOAA-NCAR twentieth century reanalysis forcing data set (He et al. 2016). This simulation has been used to estimate past OHT variability into the Arctic Ocean and thoroughly evaluated against historical hydrographic observations (Muilwijk et al. 2018).

To isolate multidecadal variability in our simulations we linearly detrended and low-frequency filtered the data with a 15-year low-pass third-order Butterworth filter for each grid point for the period 1900–2012. For estimating indices, the detrending and filtering is performed after area-averaging. Similar results are found using other cut-off frequencies. The Arctic annual mean temperature timeseries presented in Fig. 3a, b are the only data not linearly detrended.

**Fig. 2:**
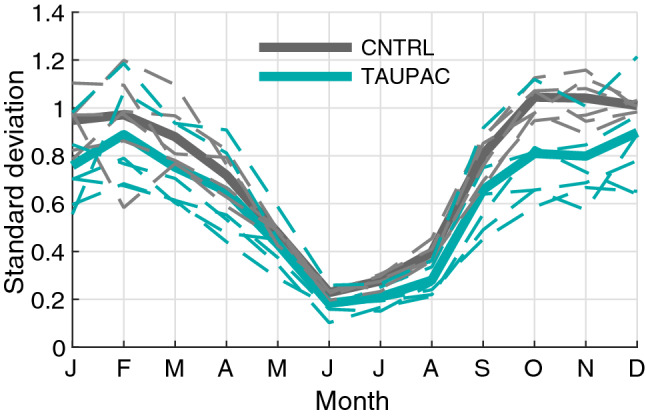
Monthly standard deviation of low-frequency filtered Arctic surface temperature in CNTRL (gray lines) and TAUPAC (green lines). Solid lines are ensemble means. Dashed lines are individual ensemble members

We focus here mostly on the cold season from October to February (ONDJF), unless otherwise stated, as this is when the decadal surface temperature variability in the Arctic is maximum in the simulations (Fig. 2), consistent with observations. Pacific variability is also maximum during boreal winter considering for instance the Aleutian Low and ENSO, as well as Pacific teleconnections (Wallace and Gutzler 1981).

## Results

### Multidecadal variability in Arctic surface temperature

**Fig. 3:**
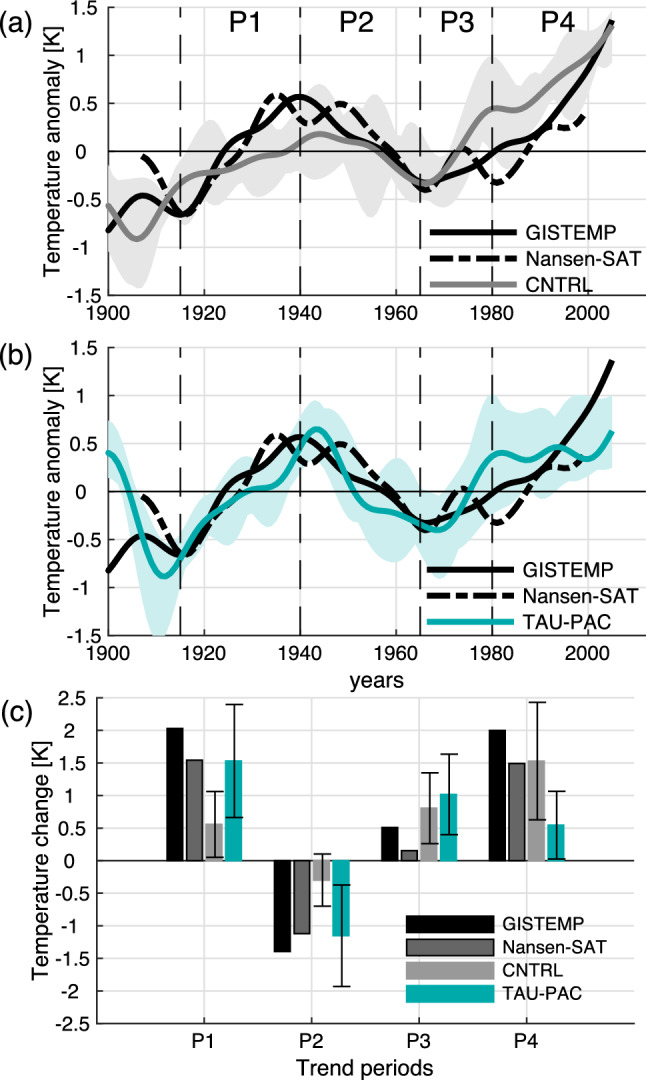
Low-frequency filtered annual Arctic Surface temperature for observational data sets (black) GISTEMP (black solid line) and Nansen-Sat (black dashed line), **a** CNTRL (gray line) and **b** TAUPAC (green line). Gray/green shading shows the ensemble spread for CNTRL/TAUPAC. The dashed vertical black lines indicate the separation of the observed warming and cooling periods P1-P4. **c** Change in ONDJF Arctic surface temperature in two observational data sets GISTEMP (black bar) and Nansen-Sat (dark gray bar with black outline), CNTRL (light gray bar) and TAUPAC (green bar), for the decadal trend periods given by the change between the average over (P1) 1911–1920 and 1936–1945, (P2) 1936–1945 and 1961–1970, (P3) 1961–1970 and 1975–1985, and (P4) 1975–1985 and 2002–2012. Error bars show the ensemble spread in CNTRL and TAUPAC

During the twentieth century, the Arctic experienced multidecadal variability in surface temperature with two periods of enhanced warming: an early warming period that lasted from around 1915 to the 1940s, a second warming period starting from the 1970s, and a cooling period in between. Figure 3 shows low-frequency filtered annual surface temperature averaged over the Arctic from 70° to 90° N for two observational data sets: GISTEMP (Hansen et al. 2010) and Nansen-SAT (Kuzmina et al. 2008). From a minimum around 1915 the Arctic surface warms by more than 1 °C until around 1940. The temperature then decreases by about 1 °C until the mid-1960s, and then increases again until present. The average temperature of the Arctic today is about 2 °C warmer than at the beginning of the twentieth century (Fig. 3).

The ensemble mean of CNTRL isolates to a degree the Arctic temperature change due to external forcing, as simulated by NorESM. The early twentieth century warming is underestimated in CNTRL [gray line in Fig. 3a and see Svendsen et al. (2018)], a common feature in many coupled climate model simulations (Wang et al. 2007). The following cooling period is underestimated as well, but in total the ensemble mean ends up at a realistic temperature anomaly at the end of the cooling period. The underestimation of the early twentieth century warming and the following cooling is also seen in the error bars in Fig. 3c for ONDJF where the temperature change in observations are clearly outside the CNTRL ensemble spread (See Sect. 3.2). The subsequent warming from 1965 is too strong until the 1980s. From the mid-1980s the Arctic starts to warm again in CNTRL. However, there are some disagreements between the observational records as well (Fig. 3), related to different sources of temperature data and interpolation methods (Kuzmina et al. 2008).

The early twentieth century warming is better reproduced in TAUPAC (Svendsen et al. 2018). The following cooling is also well simulated in TAUPAC and similar to observed, cooling more than in CNTRL (Fig. 3b). During the second warming period, the Arctic initially warms similarly in TAUPAC and CNTRL, both warming at a higher rate than observed. A clear difference between observations, CNTRL and TAUPAC is present after the 1980s as the temperature increase is weaker in TAUPAC.

In the following we investigate the different warming and cooling periods in the twentieth century in these simulations to identify mechanisms for the Arctic temperature change and the reasons for the differences between CNTRL and TAUPAC and observations. Specifically, we will compare the warming periods of 1965–1980 (P3) and 1980–2006 (P4) and the cooling period 1940–1965 (P2) with the early twentieth century warming period 1915–1940 (P1) analyzed in Svendsen et al. (2018) (dashed vertical lines in Fig. 3). These periods are chosen based on the Arctic temperature records (Fig. 3), but are somewhat consistent with the phase changes of the PDO-index as well (Fig. 1). One exception is the end point of P4, which is determined by the length of our simulations and is therefore not directly comparable with the other periods. This issue will be further discussed in Sect. 3.2.4. To investigate the changes during these four periods we look at the mean change between two 10-year periods centered around the start year and the end year of each of the periods defined above. For P1 we compare the 1936–1945 mean with the 1911–1920 mean, for P2 we compare the 1961–70 mean with the 1936–1945 mean, for P3 we compare the 1976–1985 mean with the 1961–1970 mean, and for P4 we compare the 2001–2011 mean with the 1976–1985 mean. Shifting these periods in time by a few years does not change the results qualitatively, with the possible exception of the end point of P4 for the reason noted above.

To quantify the percentage of the Pacific contribution to Arctic surface temperature change in the four periods, we assume linearity and calculate the difference in change (denoted by prefix d), where change is defined as the difference between the 10-year means defined above, in the ensemble means of TAUPAC (dTAUPAC) and CNTRL (dCNTRL) relative to the observed change (dGISTEMP) for each period P1–P4. We use the following formula: (dTAUPAC-dCNTRL)/dGISTEMP × 100. Since the ensemble sizes are small this quantification will not fully isolate the external and Pacific forced signals, and there will be uncertainty in these estimates of the Pacific contribution to Arctic surface temperature change.

### Arctic warming and cooling periods during the twentieth century

#### Early warming period 1915–1940 (P1) and a statistical link between Pacific and Arctic temperature variability

The first warming period (P1) has already been investigated in detail by Svendsen et al. (2018) using the same experiments, and here we give only a short summary of the main findings for comparison with the analysis below. Svendsen et al. (2018) showed that during the early twentieth century Arctic warming, decadal variability in the Pacific contributed to around 50% of the Arctic warming (Fig. 3c), in addition to radiative forcing. Although the ensemble size of CNTRL and TAUPAC are small leading to uncertainties in this number, the spread in estimates of temperature change in CNTRL during P1 does not include the observed temperature change, in contrast to TAUPAC (Fig. 3c). The Pacific contributed to the Arctic warming mainly through two mechanisms: firstly, advection of warm and moist air from the extra-tropics in the lower troposphere associated with a deepening of the Aleutian Low; and secondly, subsidence-induced adiabatic heating associated with a weakening of the stratospheric polar vortex, forced by anomalous atmospheric circulation induced by Pacific surface variability that strengthens upward planetary wave propagation. The influence of the Pacific on the Arctic involved a combined effect of both tropical and extratropical Pacific variability. See Svendsen et al. (2018) for more details.

**Fig. 4:**
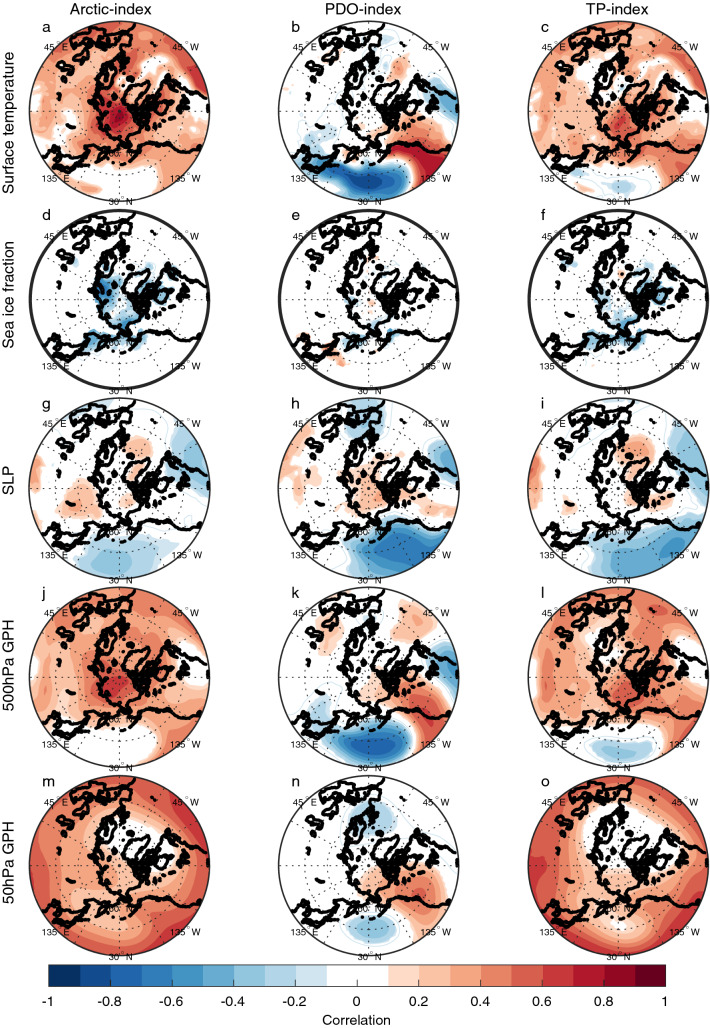
Pointwise correlation of low-frequency filtered surface temperature (**a**–**c**), sea ice fraction (**d**–**f**), SLP (**g**–**i**), 500 hPa geopotential height (**j**–**l**) and 50 hPa geopotential height (**m**–**o**) with the low-frequency filtered Arctic surface temperature index (left), PDO-index (middle) and tropical Pacific index (right) for CNTRL for the cold season ONDJF. Filled contours indicate significance at the 5% level for the effective degrees of freedom

**Fig. 5:**
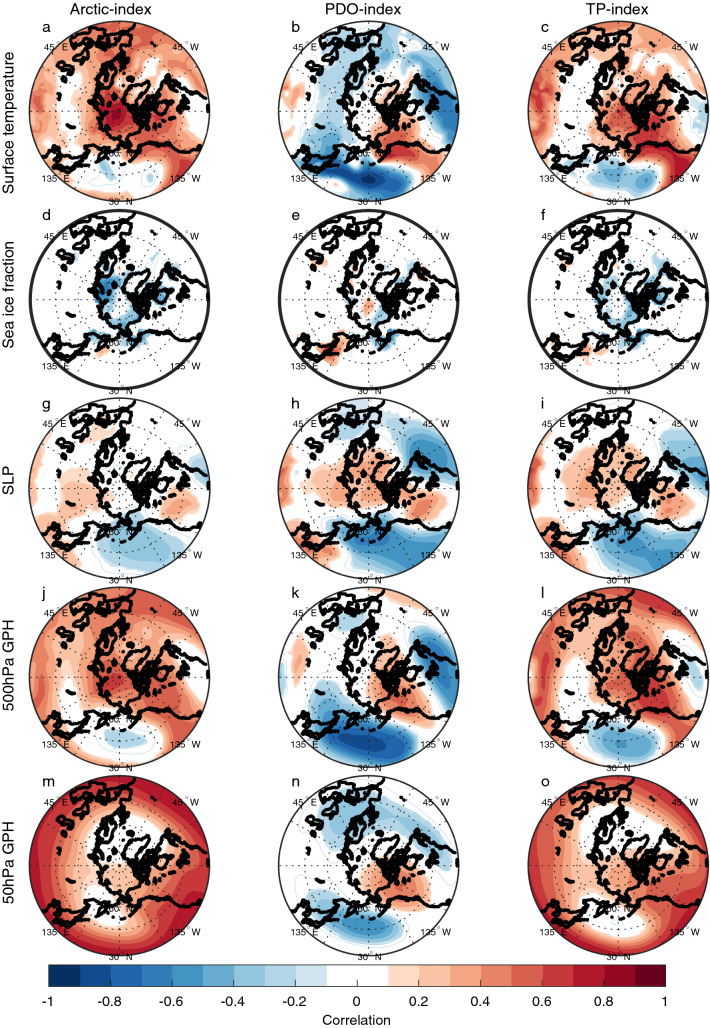
Same as Fig. 4, but for TAUPAC

The relation between the low-frequency filtered Arctic surface temperature, PDO-index and TP-index with low-frequency filtered detrended surface temperature, sea ice fraction, SLP and geopotential height is investigated in CNTRL and TAUPAC by correlation analysis (Figs. 4 and 5). The analysis reveals a relation consistent with that identified for P1. The surface temperature pattern in the Pacific related to decadal variability of Arctic surface temperature resembles a PDO pattern in both experiments (Figs. 4a and 5a). In particular, there are maximum positive correlations along the western coast of North America and in the tropical Pacific and a correlation minimum in the western North Pacific. However, the enhanced negative correlation related to the PDO (Figs. 4b and 5b) is not present for the Arctic index. There is a negative correlation between Arctic surface temperature and North Pacific SLP (Figs. 4g and 5g), indicating that a strengthening Aleutian Low coincides with warming Arctic surface temperatures. Related to this, there is a trough in geopotential height at 500 hPa in the North Pacific, and a ridge over western North America (Figs. 4j and 5j). A weaker stratospheric polar vortex is also indicated by the positive anomalies in the 50 hPa geopotential height (Fisg. 4m and 5m). These features are all consistent with the trend patterns identified during the early twentieth century warming period P1 (Svendsen et al. 2018), and suggest that similar mechanisms as found for P1 are present throughout the twentieth century.

In the Pacific half of the Northern Hemisphere, patterns of SLP and 500 hPa geopotential height associated with a positive PDO and a warm tropical Pacific are similar to those associated with a warm Arctic (Figs. 4 and 5) and consistent with the trend patterns in P1 [shown in Svendsen et al. (2018)]. These patterns are similar in CNTRL and TAUPAC, but the correlations are stronger over the Pacific region in TAUPAC in which the dynamically forced variability of the Pacific is constrained to follow observations. In the Atlantic sector, the SLP pattern associated with both the TP-index and the Arctic index in CNTRL (Fig. 4g, i) partly projects onto a negative NAO pattern together with a basin-wide surface temperature signal in the North Atlantic (Fig. 4a, c). This is reminiscent of identified links between Icelandic Low and North Atlantic SST variability on decadal-to-multidecadal timescales (Delworth and Zeng 2016; Omrani et al. 2016) as well as possible tropical Pacific teleconnection patterns (Brönnimann 2007). Inter-basin teleconnections between the North Atlantic and the tropical and North Pacific (Latif 2001; Li et al. 2016; Wu et al. 2019; Zanchettin et al. 2016; Zhang and Delworth 2007) will influence these correlation patterns. The atmospheric pattern similarities between the Arctic-index and the TP-index may also partly arise because both quantities can be independently associated with changes in the global mean temperature.

For the atmospheric variables the correlation patterns for the TP-index and the PDO-index are similar near the surface (Figs. 4 and 5h–i). But these patterns are not consistently related to the surface temperature field (Figs. 4 and 5b, c). The Arctic surface temperature is more clearly related to tropical Pacific SST than the PDO-index. Tropical SSTs impose a pan-Arctic surface temperature signal, while for the PDO the Arctic surface temperature signal is constrained to the Pacific sector of the Arctic and especially over land (Alaska and Canada). In agreement with Svendsen et al. (2018), this suggests that the combined effect of the decadal variability in the tropical and extratropical Pacific leads to a significant surface temperature response in the Arctic: a warming (cooling) tropical Pacific and negative-to-positive (positive-to-negative) phase shift of the PDO is associated with a warming (cooling) Arctic.

By introducing the wind stress anomalies over the Pacific in TAUPAC, we are somewhat changing the PDO pattern in our model. The PDO signal and associated atmospheric circulation patterns are displaced to the north in TAUPAC compared to in CNTRL (Figs. 4b, 5b). Because the wind stress anomalies we prescribe in TAUPAC come from reanalysis, we anticipate that the PDO in TAUPAC is more realistic than in the fully coupled CNTRL. Comparing the correlation patterns for the PDO-index in TAUPAC and CNTRL suggests that the Arctic response to Pacific decadal variability depends on the pattern of Pacific SST variability and its influence on the atmosphere. The next subsections will characterize the Pacific contribution to Arctic temperature trends in P2, P3 and P4.

#### Cooling period 1940–1965 (P2)

**Fig. 6:**
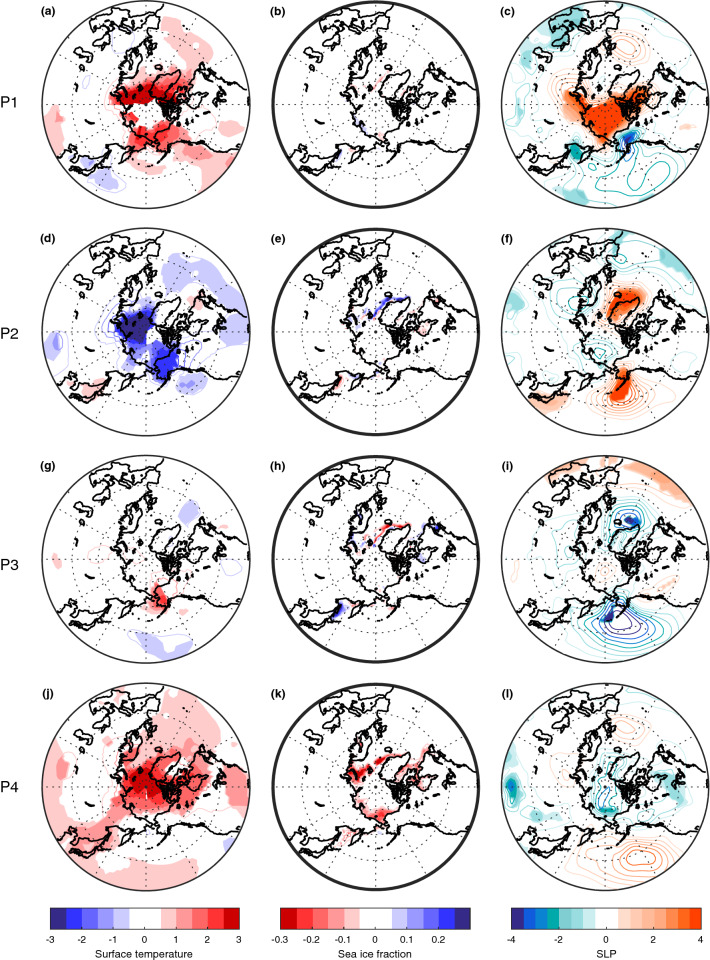
Change in observed ONDJF surface temperature (GISTEMP; left column), sea ice fraction (NSIDC data; middle column), and SLP (NOAA-NCAR 20CR; right column) for each of the decadal trend periods given by the change between the average over (P1) 1911–1920 and 1936–1945 (**a**–**c**), (P2) 1936–1945 and 1961–1970 (**d**–**f**), (P3) 1961–1970 and 1975–1985 (**g**–**i**), and (P4) 1976–1985 and 2001–2011 (**j**–**l**). Filled contours indicate significant change at the 5% level. In (**a**, **d**) there are missing values for surface temperature in the central Arctic which are marked white

**Fig. 7:**
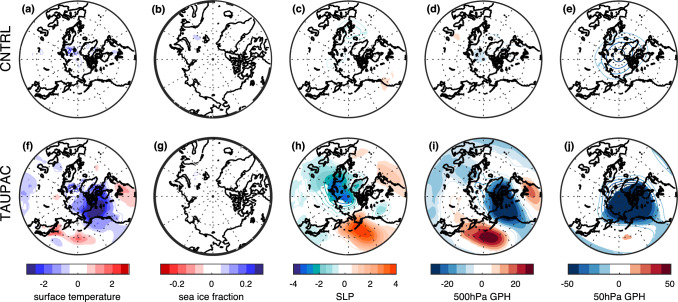
Change in ONDJF surface temperature (**a**, **f**), sea ice fraction (**b**, **g**), SLP (**c**, **h**), 500 hPa geopotential height (**d**, **i**) and 50 hPa geopotential height (**e**, **j**) for CNTRL (top row **a**–**e**) and TAUPAC (bottom row **f**–**j**) for the decadal trend period P2. Filled contours indicate significant change at the 5% level from a Student's t test based on the ensemble spread

**Fig. 8:**
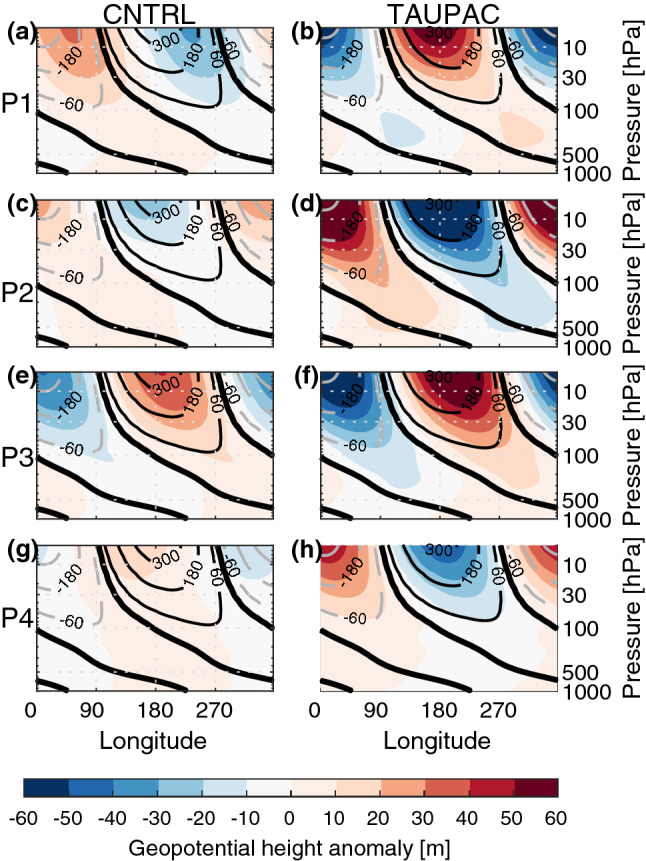
Change in the wave number 1 component (colors) of geopotential height for CNTRL (left column **a**, **c**, **e**, **g**) and TAUPAC (right column **b**, **d**, **f**, **h**) for ONDJF over the latitude band 45°–75° N for each of the decadal trend periods P1 (**a**, **b**), P2 (**c**, **d**), P3 (**e**, **f**) and P4 (**g**, **h**). Contours indicate the climatological wave number 1 and are shown at ± 60 m then for every 120 m. Thick black line indicates the zero line

**Fig. 9:**
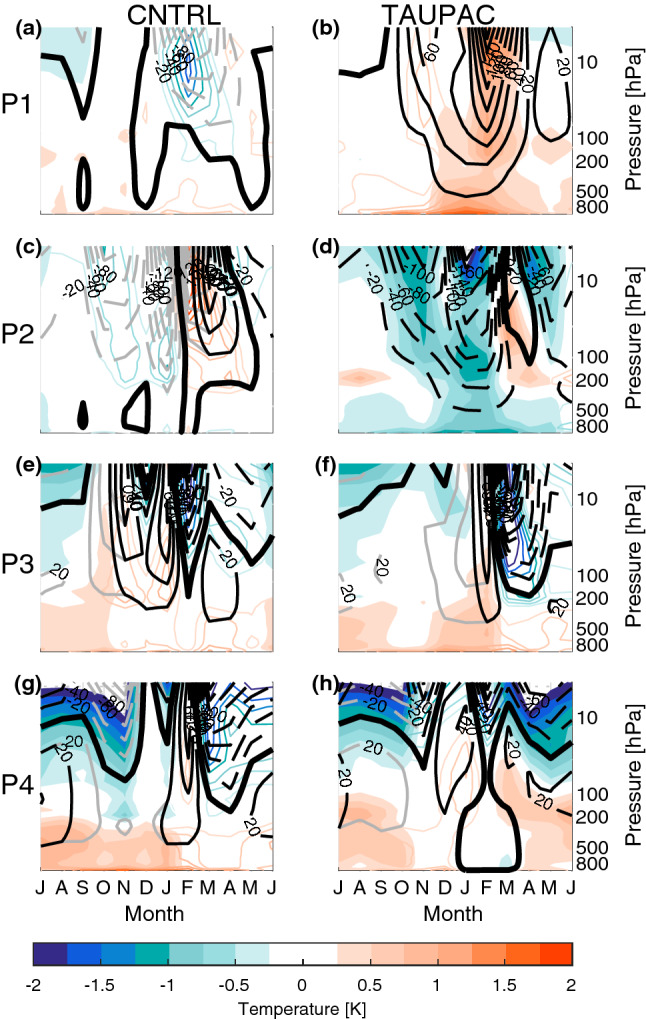
Monthly mean changes in the vertical profile of temperature (colors) and geopotential height (m, solid/dashed black or gray contours indicate positive/negative values) for CNTRL (left column **a**, **c**, **e**, **g**) and TAUPAC (right column **b**, **d**, **f**, **h**) averaged over the Arctic (70°–90° N) for each of the decadal trend periods P1 (**a**, **b**), P2 (**c**, **d**), P3 (**e**, **f**) and P4 (**g**, **h**). Filled colored contours indicate significant change in temperature at the 5% level from a Student’s t test based on the ensemble spread. Black contours indicate significant change in geopotential height at the 5% level from a Student’s t test based on the ensemble spread, otherwise the contours are gray. Thick black line indicates the 0 m geopotential height change

After the warming peak in the 1940s, the Arctic surface cools and sea ice fraction increases. Although instrumental records are still scarce during this period, the observations show a pan-Arctic cooling (Fig. 6d). The Arctic also cools significantly in TAUPAC, although more focused on the North American side (Fig. 7f) and there is no significant change in the sea ice cover (Fig. 7g). The temperature change in CNTRL is weaker and not significant (Fig. 7a) as expected from the Arctic surface temperature index (Fig. 3a). The atmospheric patterns in TAUPAC (Fig. 7h–j) are the inverse of the trend patterns for the early twentieth century warming period P1 [See Figs. 2 and 4 in Svendsen et al. (2018)], which are consistent with the correlation patterns in Figs. 4 and 5. During P2, the PDO shifts quickly from a positive phase to a negative phase (Fig. 1) with a weakening Aleutian Low (Fig. 7h) suggesting reduced moisture and heat transport towards the Arctic. During P2, there is a negative interference between the climatological and the perturbed wave number 1 (Fig. 8d) at midlatitudes between 45°–75° N, weakening the planetary wave and inhibiting upward propagating planetary waves (Fletcher and Kushner 2011). This leads to a stronger stratospheric polar vortex retaining the cold Arctic air within the Arctic, and the Arctic cools throughout the troposphere and lower stratosphere (Figs. 7j and 9d). The cooling is adiabatic and linked to large scale upward motion in the upper Arctic troposphere and stratosphere, as reflected in the negative geopotential height anomalies (Fig. 9d). This pattern is opposite of what is found in P1 (Fig. 9b). There are no significant patterns of change in the Northern Hemisphere atmosphere in CNTRL during this period (Fig. 7c–e). Overall, the Pacific contributes to ~ 55% of the surface temperature change in the ensemble mean during this period compared to GISTEMP observations (Fig. 3c, Table 1), a similar amount of explained temperature change found for the early twentieth century warming period P1. Although the ensemble size of CNTRL and TAUPAC are small leading to uncertainties in this number, the spread in estimates of temperature change in CNTRL during P2 does not include the observed temperature change, in contrast to TAUPAC (Fig. 3c), similar to the results for P1.

**Table 1:** Fractional Pacific contribution to Arctic temperature change relative to the total Arctic warming in periods P1-P4 in observations (GISTEMP), CNTRL and TAUPAC

	P1	P2	P3	P4
(dTAUPAC-dCNTRL)/dGISTEMP	0.50	0.54	0.29	− 0.44
(dTAUPAC-dCNTRL)/dCNTRL	1.34	1.40	0.15	− 0.60
(dTAUPAC-dCNTRL)/dTAUPAC	0.53	0.58	0.13	− 0.60

#### Second warming period 1965–1980 (P3)

From around 1965, the Arctic surface warms again (Fig. 3). At the same time the PDO shifts from a negative to a positive phase again (Fig. 1). During P3, both CNTRL and TAUPAC simulate a similar degree of warming; they overestimate the observed warming in their ensemble means, although the (GISTEMP) observations mostly lie within the ensemble spread (Fig. 3). In contrast to P1, during ONDJF there is hardly any significant tropospheric warming in P3 in CNTRL, while the significant Arctic warming is confined to the troposphere in TAUPAC; also, geopotential height anomalies do not show stratospheric induced subsidence and heating over the pole (Fig. 9e, f). This may suggest the impact of radiative forcing and surface feedbacks. The similarities between the two ensembles also imply that external forcing could be the dominant reason for the Arctic warming in P3.

**Fig. 10:**
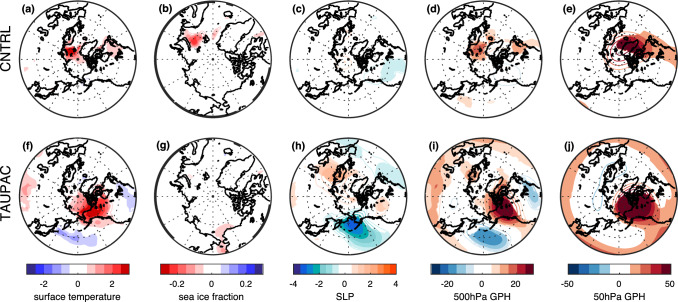
Same as Fig. 7, but for the decadal trend period P3

Even though the total Arctic warming in CNTRL and TAUPAC are comparable during P3, there are some regional differences in the Arctic warming pattern between CNTRL and TAUPAC (Fig. 10a, f). This is possibly related to the Pacific-forced atmospheric circulation changes in TAUPAC that can determine the distribution of heat within the Arctic. The observations show that during P3 the Arctic warms mainly around the Bering Strait (Fig. 6g). In TAUPAC this pattern is reproduced although somewhat enhanced (Fig. 10f). For TAUPAC, the atmospheric circulation trends in P3 are similar to P1, although the signal over the Arctic is more confined to the Pacific and North American side of the Arctic (cf. Fig. 2 of Svendsen et al. 2018). As during P1, and similar to observations (Fig. 6i) the Aleutian Low is deepening (Fig. 10h). A positive inference between the background and anomalous Wave number 1 in midlatitude geopotential height (Fig. 8f) weakens the stratospheric polar vortex (Fig. 10j) similar to in P1, although the polar vortex response is more confined to the Pacific and North American side of the Arctic.

**Fig. 11:**
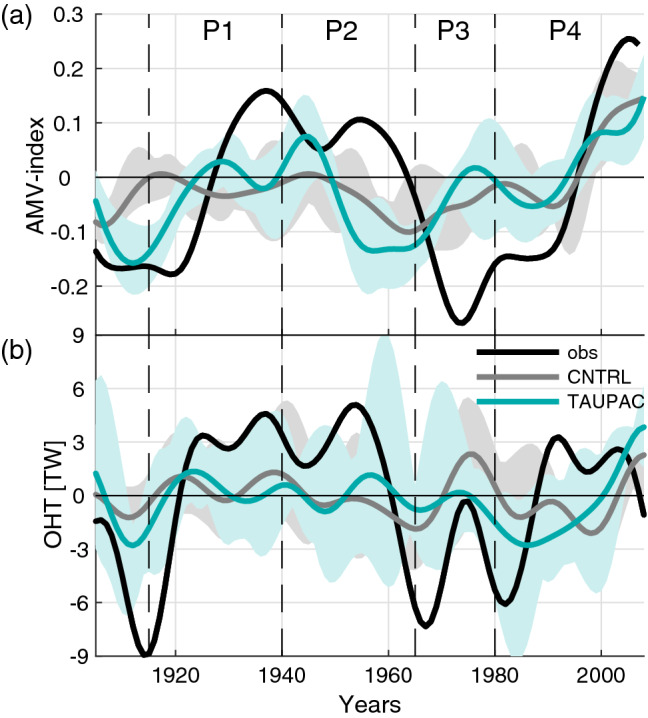
**a** AMV-index from HadISST (black line), CNTRL (gray line) and TAUPAC (green line), and **b** ocean heat transport (OHT) through the Barents Sea Opening in CNTRL (gray line), TAUPAC (green line), and an observed estimate (black line) from Muilwijk et al. (2018). Gray/green shading shows the ensemble spread for CNTRL/TAUPAC

Contrastingly, in CNTRL there is no change in the Aleutian Low (Fig. 10c). The Arctic warms mainly in the Barents Sea, on the Atlantic side of the Arctic (Fig. 10a). In CNTRL, the warming in the Barents Sea area coincides with the area of sea ice loss (Fig. 10b) and the surface atmosphere warming could be related to longwave radiative forcing and surface heat fluxes from below. Diabatic heating contributes more to the increased near-surface Arctic temperature in CNTRL compared to TAUPAC during P3 (not shown), consistent with reduced sea ice in the area (Higgins and Cassano 2009). The OHT through the Barents Sea Opening in CNTRL is also positive in P3 in contrast to in TAUPAC and observational estimates (Fig. 11b), consistently contributing to the sea ice loss and warming in this region in CNTRL (see Sect. 3.3).

In summary, the impact of the Pacific on the Arctic temperature trend during P3 is through similar mechanisms as found for earlier periods, but the contribution to the total warming is less as it is constrained to the Pacific side of the Arctic. Variability in the Pacific contributes to only ~ 15% additional warming in TAUPAC when comparing with CNTRL (Fig. 3c, Table 1). This implies that external forcing is the main contributor to the Arctic warming in P3 in our simulations. However, the relative importance of Pacific versus external forcing is more uncertain than for periods P1 and P2, as both TAUPAC and CNTRL ensembles contain the observed Arctic surface temperature change and the differences between CNTRL and TAUPAC could be due to internal variability outside of the Pacific region. The differences in the warming patterns as well as circulation changes between CNTRL and TAUPAC suggest complex interactions between the radiative signal and the Pacific-forced changes, but the warming and circulation patterns in TAUPAC resemble the observations more than CNTRL does.

#### The period 1980–2006 (P4)

**Fig. 12:**
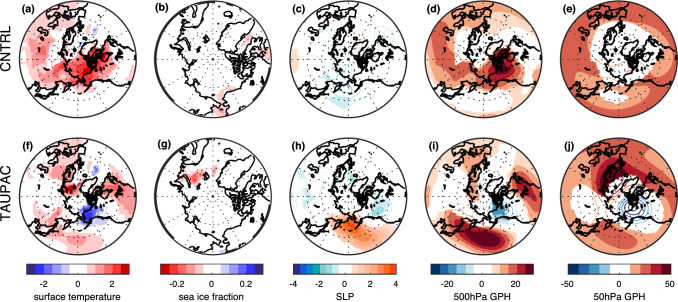
Same as Fig. 7, but for the decadal trend period P4

During the last decades of the simulations, Arctic temperatures continue to increase (Fig. 3). TAUPAC underestimates the rate of Arctic warming (Fig. 3c). Overall, CNTRL achieves a final temperature anomaly close to observations, while TAUPAC is ~ 0.5 °C too cold. Observations show a pan-Arctic warming, albeit weaker around the Bering Strait (Fig. 6j). However, the temperature change in this region is not robust among different reanalysis products (Lindsay et al. 2014). In CNTRL the warming extends south into Alaska, Canada and Northern Russia (Fig. 12a). In contrast, Alaska and the Bering Strait region are cooling in TAUPAC, while the Atlantic side of the Arctic is warming (Fig. 12f). Consistent with a positive-to-negative phase shift of the PDO, there is a weaker Aleutian Low (Fig. 12h), but no significant response in the polar stratosphere (Fig. 12j). The Pacific impact on the Arctic is limited to the troposphere in P4. The warming in CNTRL is confined to the lower and middle troposphere with cooling aloft, and in CNTRL and TAUPAC the geopotential height field does not indicate stratospheric connected adiabatic warming at upper levels (Fig. 9g, h); this is indicative of radiative forced warming and surface feedbacks, as well as low-level atmospheric heat advection.

**Fig. 13:**
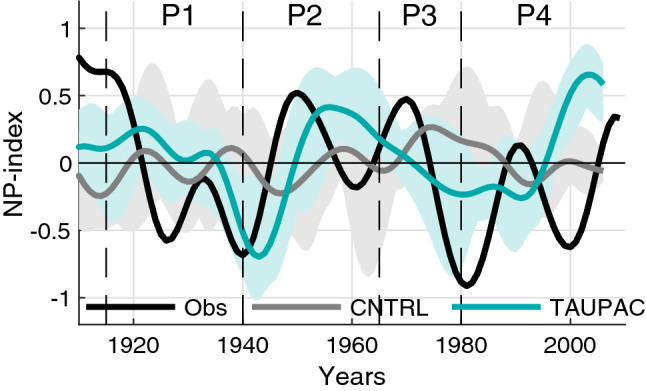
NP-index from observations (Trenberth and Hurrell 1994; black solid line), CNTRL (gray line) and TAUPAC (green line) for the cold season ONDJF. Gray/green shading shows the ensemble spread for CNTRL/TAUPAC

As stated above, the Aleutian Low weakens in TAUPAC during P4 (Fig. 12h), but this signal is not significant in observations (Fig. 6l). This leads to reduced heat and moisture transport into the Arctic in P4 in TAUPAC limiting the Arctic warming. While the other periods investigated here, P1-P3, are defined by the Arctic surface temperature trend rates and fit well with the PDO tendencies, the end of P4 is determined by the end of our simulations and the length of the NOAA-NCAR twentieth century reanalysis product (Compo et al. 2011) used to constrain simulated Pacific variability. The end of P4 is therefore centered around year 2006 which is in the middle of a negative PDO phase and the Arctic temperatures are still increasing. This issue of endpoint could be important for the Aleutian Low variability (Fig. 13) since the timing of Aleutian Low variations in P4 are shifted compared to observations. Future simulations with an updated reanalysis product will help clarify this.

During P4, the cooler Pacific is compensating for the radiative forced Arctic warming around the Bering Strait, in total contributing to reducing the Arctic temperature change by ~ 45–60% depending on the reference data and keeping in mind the ensemble spread (Fig. 3c). A quantification of the Pacific contributions to the simulated Arctic temperature change in each period is summarized in Table 1.

In short, TAUPAC underestimates the Arctic warming in P4 compared to GISTEMP, and overestimates the warming in P3. While CNTRL tends also to underestimate the GISTEMP warming, it agrees better with the observations, which lie within the ensemble spread. However, the surface temperature and SLP patterns in TAUPAC resemble observations more closely than in CNTRL. There seems to be a component that impacts Arctic temperature that is not included in either CNTRL and TAUPAC that could be of specific importance for P3 and P4. The following argues the role of the Atlantic during these two last periods.

### Possible contribution from the Atlantic

As mentioned in the introduction, multidecadal variability of North Atlantic SST has also been linked to Arctic temperature trends. For instance, Tokinaga et al. (2017) found similar weights for both the Pacific and the Atlantic contribution to the early twentieth century Arctic warming. In P1 and P2 the Pacific can explain a majority of the Arctic decadal surface temperature trends that cannot be explained by external forcing (determined by the CNTRL ensemble keeping in mind the uncertainties related to internal variability for the small ensemble size). However, this is not the case for P3 and P4, where the impact of Pacific variability contributes to an underestimation in P4 of the Arctic temperature change in TAUPAC, while both TAUPAC and CNTRL seem to overestimate the warming in P3 compared to observations. In reality, it is therefore likely that something could be counteracting the impact of the Pacific, and the following results suggest that Atlantic variability is playing a part.

In our ensembles, the internal multidecadal variability of the Atlantic is not dynamically synchronized with observations. An estimated external forced part of the AMV can be seen as the ensemble mean of CNTRL (Fig. 11a), although internal variability will still be present in the mean of the small ensemble. While there may be a Pacific-forced part of the AMV, such a component is not obvious in our simulations, as there is little difference between the ensemble means of TAUPAC and CNTRL (Fig. 11a). The AMV variability is significantly underestimated in the simulations (according to a one-tailed F-test using a 95% confidence level taking into account individual ensemble members), and the influence of the Atlantic on the Arctic could be underestimated in the model. The standard deviation of AMV in observations is 0.14 K, while it is 0.07 K for CNTRL (ensemble spread: 0.06–0.09 K) and 0.09 K for TAUPAC (ensemble spread: 0.09–0.12 K). The Atlantic Ocean temperatures can induce atmospheric teleconnections that effect the Arctic (Castruccio et al. 2019) and can impact the Arctic directly through OHT into the Barents Sea and through the Fram Strait. A similar phasing of observed AMV and low-frequency filtered OHT through the Barents Sea Opening can be seen by comparing Fig. 11a and b, but the multidecadal signal is clearer in the AMV index. Multidecadal variability in OHT is not clearly apparent in TAUPAC nor CNTRL, indicating it might be unrelated to external forcing.

Of specific interest is the phasing of AMV anomalies during P3 and P4 (Fig. 11). During P3 the observed AMV was strongly negative with cool SSTs in the North Atlantic and negative Atlantic OHT anomalies. In TAUPAC and CNTRL, this is not the case. The North Atlantic is actually warming in both TAUPAC and CNTRL. The OHT through the Barents Sea Opening is also positive in CNTRL leading to the warming in the Barents Sea, which can perhaps account for the enhanced Arctic warming in CNTRL compared to observations during P3 (Fig. 3c). During P3, the warming Atlantic in CNTRL adds to the externally forced warming in the Arctic. In TAUPAC, the OHT is neutral, while in observations the negative OHT anomalies from the Atlantic are counteracting the externally forced and the Pacific-forced Arctic warming. In total, CNTRL and TAUPAC overestimate the Arctic surface warming during P3 mainly because of discrepancies between simulated and observed AMV and Atlantic OHT.

During P4, we find the opposite North Atlantic anomalies compared to P3. Observations show a strong warming of the North Atlantic surface (Fig. 6j), and historical estimates indicate a consecutive increase in OHT through the Barents Sea Opening (Fig. 11b) with a clear negative-to-positive phase shift of the AMV (Fig. 11a). The increased OHT from the Atlantic into the Arctic Ocean through the Barents Sea Opening in observations may contribute to the warming of the Arctic and counteracts the cooling response from the Pacific. In TAUPAC and CNTRL the AMV-index is already neutral in the beginning of P4 and OHT anomalies are mostly negative, consistent with the underestimation of Arctic warming in both simulations. In addition, in TAUPAC the negative PDO phase further offsets the externally forced warming, and as a result TAUPAC strongly underestimates the warming of the Arctic in P4 (Sect. 3.2.4).

**Fig. 14:**
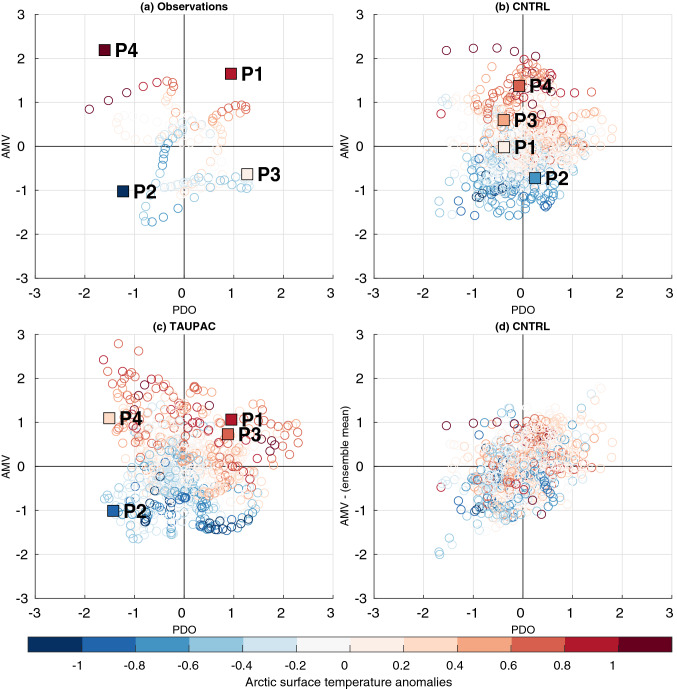
Scatter diagrams of Arctic surface temperature anomalies (circles) as a function of normalized AMV and PDO indices in **a** observations **b** CNTRL and **c** TAUPAC. The filled squares indicate the mean change in Artic temperature as a function of the change of AMV and PDO for each of the 4 trend periods P1-P4. **d** Scatter diagram of Arctic surface temperature anomalies as a function of normalized AMV and PDO indices in CNTRL, where the ensemble mean of CNTRL has been removed from the AMV index in each ensemble member to eliminate the external forced part of the AMV. For the TAUPAC and CNTRL every year in every ensemble member is considered, identifying the value of the low-frequency filtered Arctic surface temperature anomaly and the corresponding values of the AMV and PDO indices

The relative roles of Pacific decadal variability, North Atlantic temperature anomalies and external forcing are further illustrated in the scatter diagrams of low-frequency filtered Arctic surface temperature as a function of the AMV and PDO indices (Fig. 14). For the observations, warm (cold) conditions in the Atlantic and Pacific are consistent with warm (cold) conditions in the Arctic (Fig. 14a). For TAUPAC where the PDO is simulated as observed, the Arctic temperature varies with both PDO and AMV (Fig. 14c), with similarity to observations. For CNTRL (Fig. 14b), the Arctic temperature seems to be determined by the AMV-index. However, we have to keep in mind that the AMV here could be a combination of external forcing and internal variability (Otterå et al. 2010), and the external forcing is a common driver for both the Atlantic and the Arctic temperatures. The link between AMV and Arctic surface temperatures in CNTRL can be partly explained by the correlation with the common external forcing. This is clarified in Fig. 14d where we have removed the ensemble mean of the AMV in every ensemble member of CNTRL to eliminate the externally forced part of AMV. When doing this, the Arctic surface temperature anomalies seem to be related to the sign of both the AMV and the PDO, but there remains a large scatter with warm (cold) Arctic conditions also coinciding with cold (warm) PDO in some instances (Fig. 14d). Consequently, we should consider the AMV-index in Fig. 14a–c to be a combination of externally forced and internally driven North Atlantic SST variability. It should also be noted that the PDO and AMV indices are not necessarily independent and possible inter-basin interactions exist (Latif 2001; Zhang and Delworth 2007).

The squares in the scatter diagrams in Fig. 14a–c summarize the changes in Arctic temperature, AMV (including external forcing) and PDO for P1–P4, and illustrate the roles of the Pacific and Atlantic (including external forcing) for the decadal trends in the Arctic. For TAUPAC the boxes are in the same quadrant as for observations except for P3. For P1, TAUPAC is in the first quadrant with both a positive AMV and positive PDO. Observations and TAUPAC are largely consistent, indicating that including external forcing and the Pacific variability, gives a consistent warming in the Atlantic and simultaneously an early twentieth century warming of the Arctic. For P2, observations and TAUPAC are in the opposite situation, in the third quadrant, with a negative tendency in PDO and AMV and the external forcing signal, leading to a cooling Arctic. For P3 both CNTRL and TAUPAC overestimate the observed Arctic warming (See Fig. 3c and color of P3 squares in Fig. 14). As stated above, the North Atlantic is too warm for both TAUPAC and CNTRL during P3, so TAUPAC is in the first quadrant as in P1, but observations are in the fourth quadrant (negative tendency of AMV, positive tendency of PDO). AMV can be seen as a proxy for OHT from the Atlantic, but AMV and OHT from the Atlantic are not equivalent. Replacing AMV with OHT in Fig. 14 would place TAUPAC closer to observations since the OHT was neutral for TAUPAC during P3. For P4, both observations and TAUPAC are in the second quadrant (negative tendency of PDO, positive tendency of AMV), but the Atlantic signal in TAUPAC (and CNTRL) is too weak, and the magnitude of the Arctic warming is too small, as concluded above.

## Discussion

We have found that the decadal variability in the Pacific can influence Arctic surface temperature, and may have had considerable impact on Arctic surface temperature trends throughout the twentieth century. Our results and previous studies indicate that the Pacific impact can differ depending on the region of the Arctic, related to geographical proximity to the Pacific and if the region is ice covered or not (Meehl et al. 2018; Screen and Deser 2019). Surface temperature change during winter for sea ice covered areas are mainly caused by atmospheric heat advection and possibly adiabatic heating, while in areas with no or little sea ice the temperature change also depends on sea ice drift and processes related to evaporation and longwave radiation (Graversen et al. 2011; Lee et al. 2011; Meehl et al. 2018; Screen and Deser 2019; Svendsen et al. 2018).

The temperature response to northward atmospheric heat and moisture advection from a stronger Aleutian Low is mainly detected around the Pacific side of the Arctic. The impact of the Atlantic on Arctic surface temperatures and sea ice cover, has been mainly linked to the Atlantic side of the Arctic through OHT through the Fram Strait and Barents Sea Opening (Årthun et al. 2012; Bengtsson et al. 2004; Smedsrud et al. 2013). Meehl et al. (2018) found that trends in tropical convective heating, consistent with a cooling tropical Pacific as in P2 and P4, can also lead to less sea ice in the Atlantic sector of the Arctic through sea ice drift. This link seems not to be dominating in the earlier cooling period P2, but could be at work in P4. The presence of this statistical link between tropical Pacific cooling and Barents Sea warming in the later part of the century compared to the first part, could be related to the mean sea ice extent and thickness. The mean sea ice extent has been decreasing considerably since the 1970s (e.g. Serreze et al. 2007). Since decreasing sea ice extent is associated with increased kinematics the sea ice in the later period could be more sensitive to wind-driven sea ice drift (Rampal et al. 2009). However, most CMIP climate models, including NorESM1-ME used here, do not capture sea ice drift acceleration (Rampal et al. 2011), and we do not reproduce the same atmospheric SLP pattern over the Atlantic-Arctic as identified in Meehl et al. (2018) in P4 either. The Barents Sea temperature and sea ice trends in TAUPAC are less directly related to decadal variability in the Pacific than the rest of the Arctic, consistent with Screen and Deser (2019). The mechanism for the warming Barents Sea in P4 in TAUPAC is therefore related to a different mechanism than proposed by Meehl et al. (2018). One likely candidate could be Atlantic OHT (Fig. 11b) or local internal atmospheric variability. However, simulated regional temperature anomalies might be more sensitive to the model’s climatological sea ice extent and thickness than large-scale atmospheric circulation patterns, compared to the pan-Arctic perspective.

Another regional feature is the surface warming trends over Greenland. Neither TAUPAC or CNTRL reproduce the observed warming over Greenland in P4. Ding et al. (2014b) proposed that the negative tropical Pacific SST trend in recent decades forced a Rossby wave train across North America to the Atlantic, resulting in a warming over Greenland and north-eastern Canada. The teleconnection pattern found in Ding et al. (2014b) is not present in our model in any period (Figs. 4 and 5), but the teleconnection pattern identified by Ding et al. (2014b) can be displaced in climate models (Ding et al. 2018). On the other hand, McCrystall et al. (2020) found the opposite atmospheric response from tropical Pacific SST perturbations. The observed Greenland surface temperature trend could also be forced by local SSTs related to sea ice loss, rather than remotely forced by the Pacific (Fumiaki Ogawa, personal communication). Regardless, the Greenland surface temperature trends contribute minimally to the pan-Arctic warming trends. By removing Greenland in our calculations of Arctic surface warming, the temperature change difference between our simulations and observations reduces by less than 0.1 K in P4. Thus, the underestimation of Greenland warming in TAUPAC does not contribute substantially to the overall underestimation of warming in P4.

**Fig. 15:**
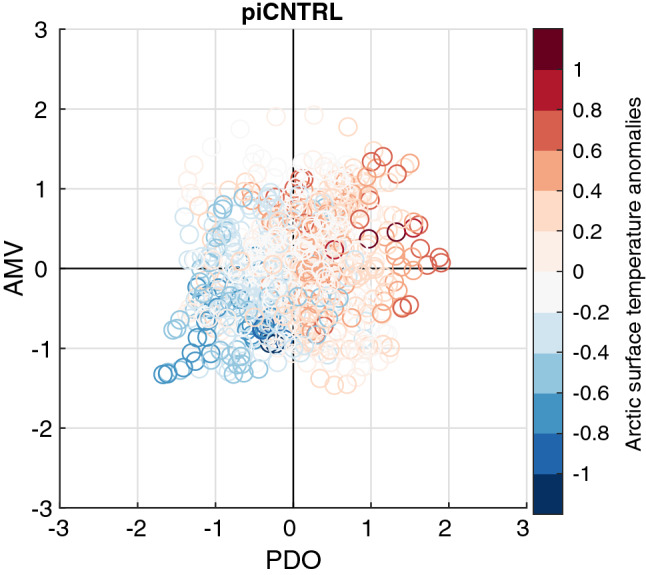
Scatter diagram of Arctic surface temperature anomalies as a function of normalized AMV and PDO indices in piCNTRL of NorESM

In the present study we have quantified the relative contribution of the Pacific influence on decadal Arctic temperature trends during the twentieth century. However, the percentage of contribution will likely depend on the model’s climate sensitivity. Tokinaga et al. (2017) found comparable contributions from the Atlantic and the Pacific on Arctic surface temperature in a suite of CMIP5 pre-industrial control simulations with constant external forcing. Compared to CMIP5 pre-industrial simulations, NorESM may be among the models where the Pacific is a stronger determinant for Arctic surface temperature than the Atlantic on decadal timescales. In piCNTRL, Arctic surface temperatures seem to be more strongly related to the PDO-index than the AMV-index (Fig. 15). One reason for this could be that NorESM might overestimate the impact of the Pacific through the response of the Aleutian Low. The simulated amplitude of the Northern Annular Mode (NAM) in the Arctic and North Pacific is larger than observed and explains 36% of the NH atmospheric variability compared to 25% in observations, a common bias in CAM-based atmospheric models (Bentsen et al. 2013). Another reason could be the underestimation of the AMV amplitude in NorESM, with a standard deviation of 0.05 K in the preindustrial control simulation, compared to the observed 0.14 K (Bentsen et al. 2013). However, our analysis shows that this bias might be more important during the last half of the twentieth century when Arctic sea ice is reduced (P3 and P4) than during P1 and P2 as TAUPAC reproduces the observed temperature trends in P1 and P2, while discrepancies exist in P3 and P4.

The comparison of P1 with P3 suggest that the relative strength of the Pacific-Arctic teleconnection pattern depends on the strength of the radiative forcing and regional sea ice extent. The summer melting rate in NorESM is low and the mean sea ice is too thick (Bentsen et al. 2013). It seems likely that this could impact the extent of the Arctic surface temperature response to external radiative forcing related to sea ice feedbacks. At the same time, NorESM simulates tropical variability well and may therefore better simulate the teleconnection patterns from the tropics (Bellenger et al. 2014; Sperber, 2013). An in-depth inter-model comparison is the focus of ongoing work.

Lastly, the Pacific variability is reproduced in TAUPAC by prescribing momentum flux anomalies from reanalysis (Compo et al. 2011), and data are especially scarce over the North Pacific before the 1950s. In addition, there are large uncertainties in Arctic climate before the satellite era starting in 1979, limiting the confidence in our comparison with observations before the 1980s. Regardless, the results presented here provide a possible scenario for the historical Pacific impact on Arctic surface temperature trends.

## Summary and conclusions

In this study, we have investigated the impact of decadal variability in the Pacific on Arctic surface temperature and atmospheric circulation trends during the twentieth century. We have used coupled model simulations including transient external forcing where we in addition have phased Pacific variability to observed. We find that the Pacific can contribute up to ~ 50% of the decadal temperature trends in the Arctic. Our simulations also show that the impact of the Pacific on Arctic temperature trends is symmetric in the sense that the Arctic atmosphere response to negative anomalies in the Pacific are mirrored and opposite of the response to positive anomalies, i.e. the atmospheric trend patterns are opposite in P1 and P2.

The Pacific impact on the Arctic is a result of the combination of tropical and extratropical Pacific variability. Tropical Pacific SSTs impact the Arctic further north via atmospheric wave-train teleconnection mechanisms, while the extratropical Pacific impact, quantified using the PDO-index, is more constrained to the North American side of the Arctic. However, the exact extent of the extratropical Pacific impact on the Arctic depends on the PDO pattern. We find that when we constrain Pacific variability to observed in TAUPAC, the PDO has a more Pan-Arctic impact compared to in CNTRL.

The lack of realistic anomalies in the Atlantic sector in our simulations might inflate the Pacific impact during later periods when sea ice is reduced and external forcing is stronger. The role of the Atlantic is difficult to separate from the external forced signal, as both external forcing and multidecadal Atlantic SST variability have global impacts (Knight et al. 2006; Sutton and Hodson 2007; Ting et al. 2011; Zhang and Delworth 2006) and Atlantic SSTs and external forcing are to some extent in phase (Booth et al. 2012; Otterå et al. 2010; Vecchi et al. 2017). The fact that the AMV seems to have an external forced part in our simulations might also inflate the apparent impact of AMV on the Arctic surface temperature. The present results indicate that both external forcing and Atlantic variability can reinforce or depreciate the Pacific influence, especially in the last decades of the twentieth century when external forcing has increased and sea ice cover has reduced. Analysis of the Pacific impact on the Arctic in an unforced system, is the focus of our ongoing research.

Our results indicate that the changes in the Pacific in recent decades associated with a negative PDO counteracted the externally forced warming over the Arctic. These results therefore have implications for decadal predictability of the Arctic. If the PDO stays positive for the next decades, we expect an accelerated warming in the Arctic, at least around the Bering Strait and eastward. Indeed, there is evidence of record low sea ice extent around the Bering Strait in recent winters of 2017/2018 and 2018/2019 (https://nsidc.org/arcticseaicenews/). The Pacific influence on the Arctic may also have become stronger since 2007 (Yang et al. 2020). However, the full extent of the warming trends will depend on the development of North Atlantic anomalies and the character of future external forcing. A pan-Arctic impact of the Pacific will likely also depend on the SST trend pattern in the Pacific. At the moment, unfortunately, there are few promising studies demonstrating decadal predictability of Pacific decadal variability (Newman et al. 2016), and the influence of the Pacific on the Arctic may rather reduce the predictability of near-term changes.

## Data Availability

The data from the model simulations of NorESM that support the findings of this study are publicly accessible through the Research Data Archive of UNINETT Sigma2.

## Code availability

The modifications of NorESM made to perform the partial coupling experiments are available from the corresponding author on request.
